# 896. Examining the Impact of the COVID-19 Pandemic on Delivery of HIV Care and Prevention Services Among Patients in a Ryan White Clinic

**DOI:** 10.1093/ofid/ofab466.1091

**Published:** 2021-12-04

**Authors:** Michelle Zhang, Sharlay Butler, Jason Kennedy, Molly McKune, Ghady Haidar, Deborah McMahon

**Affiliations:** 1 University of Pittsburgh School of Medicine, Pittsburgh, Pennsylvania; 2 UPMC, Pittsburgh, Pennsylvania; 3 University of Pittsburgh Graduate School of Public Health, Pittsburgh, Pennsylvania; 4 University of Pittsburgh Medical Center, Pittsburgh, PA; 5 University of Pittsburgh, Pittsburgh, Pennsylvania

## Abstract

**Background:**

We sought to characterize the impact of the COVID-19 pandemic on HIV-related outcomes in a cohort of patients by examining rates of viral load (VL) suppression, retention-in-care, PrEP access, and STIs.

**Methods:**

This was a single center, retrospective study of adults receiving HIV treatment or HIV/STI prevention services from 01/2019 - 12/2020. HIV outpatient visits were identified through HRSA’s CareWARE. Visits (in-person, telehealth) only included HIV primary care. HRSA core performance measures were utilized (Table 1). STI positivity rates and descriptive characteristics were calculated. New and refill PrEP prescriptions were tabulated. Chi-square tests compared unmatched non-parametric variables; McNemar’s test matched non-parametric variables. Multivariable logistic regression identified variables associated with retention in care and viral suppression.

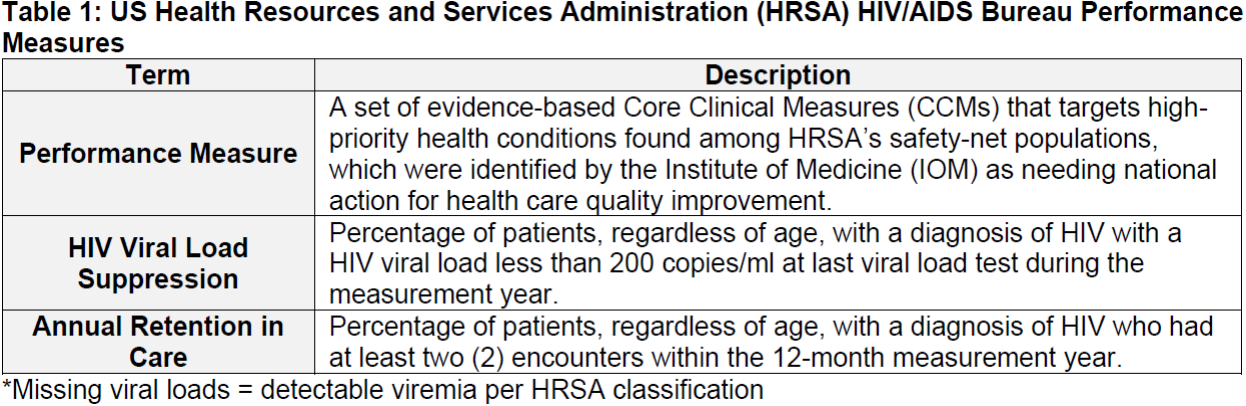

**Results:**

1721 patients received care; 1234 were seen in both years, 334 only in 2019, 153 only in 2020. The number of telehealth visits increased significantly: video (0% to 31%, < 0.001), phone (0% to 0.4%, p < 0.001). Though the proportion of kept appointments increased (57.2% vs 61.2%), the annual retention in care rate decreased from 74.5% to 70.9% (p = 0.002). Overall, 9.7% of patients had detectable VLs at any point. Compared to 2019, a lower proportion of patients maintained VL suppression in 2020, (91.6% vs 83.5% p = 0.075). More patients did not have a VL drawn in 2020 than in 2019 (10.3% vs 2.0 %, p < 0.001). Patients with detectable VLs in 2019 were more likely than those who were undetectable to have detectable VLs in 2020 (OR 18.2, 95% CI 9.91-33.42). Black race was associated with higher likelihood of lack of VL suppression (OR = 2.0; 95% CI 1.10-3.66). There were no significant differences between gender or age groups in rates of viral suppression, number screened for bacterial STIs or positive results. Visits for new and refill PrEP prescriptions decreased by 59% and 7%, respectively.

**Conclusion:**

Rates of viral load suppression and retention in care decreased in 2020 compared to 2019. The proportion of clinic visits attended increased after the integration of telemedicine in 2020. These data may be used to inform evidence-based interventions to improve the HIV continuum of care through telehealth.

**Disclosures:**

**Ghady Haidar, MD**, **Karuys** (Grant/Research Support)

